# Supplementary material for: Are carbon emissions trading and green financial instruments synergistic? -Comprehensive quantitative research based on content analysis
Source: PLoS One. 2024 Mar 7;19(3):e0298601. doi: 10.1371/journal.pone.0298601 (PMC10919601; doi:10.1371/journal.pone.0298601)
Supplement: S1 Data — (ZIP) [file pone.0298601.s001.zip › Data/地级市Z_F工作报告.zip!/┴╔─■/╔≥╤⌠╩╨/└┤╫╘╣1⁄2╓┌║┼í╛┬φ┐╦╩2╛▌═°í┐_2006.docx]

各位代表，同志们：

　　现在，我代表市人民政府向大会作工作报告，请予审议，并请各位政协委员提出意见和建议。

　　一、2005年和“十五”时期政府工作简要回顾

　　2005年，在省委、省政府和市委的领导下，在市人大的依法监督和市政协的民主监督下，市政府带领全市人民高举邓小平理论和“三个代表”重要思想的伟大旗帜，坚持科学发展观，同心同德，努力奋斗，在沈阳老工业基地振兴中迈出了新的步伐。

　　预计全市地区生产总值完成2240亿元，同比增长16％；全社会固定资产投资实现1363.2亿元，增长40.3％；社会消费品零售总额实现915.1亿元，增长13.1％；地方财政收入实现181.5亿元，增长30.1％，连续五年保持30％以上的速度递增；财政支出完成250.9亿元，增长23.4％；城市居民人均可支配收入达到10098元，增长13.2％；农民人均纯收入达到5050元，增长16.2％。

　　过去的一年，我们实现了工业经济的高速增长，基本完成了世园会筹备工作，期盼多年的地铁工程终于开工建设。这些成果标志着沈阳各项事业攀上了一个新的高峰，鼓舞着720万人民万众一心，去创造更加美好的未来！

　　（一）“工业年”活动硕果累累，工业整体规模和水平大幅提升

　　2005年，全市规模以上工业总产值完成2288.6亿元，同比增长39.3％；实现工业增加值624.3亿元，增长40.5％；工业固定资产投资完成510.3亿元，增长76.7％；工业经济效益综合指数达到141.3％，同比提高9.4个百分点。

　　全市新开工建设投资500万元以上工业项目1145个，总投资705亿元，当年已竣工投产的项目766个。项目全部达产后，创造工业总产值将超过1900亿元，相当于再造一个沈阳规模以上工业。全市规模以上工业企业由2004年末的1836户增加到3033户。规模以上工业总产值在15个副省级城市中的排名，由1999年的第13位上升到去年的第8位，这种良好的势头还在继续发展。

　　培育和引进“三个20”年销售收入过百亿元的大项目和产业集群是沈阳工业实现跨越式发展的关键，现已有8户企业和7个产业集群初具规模，经过几年的努力，我们的规划将会变成现实。

　　国有企业战略性重组的最后攻坚战全面展开，24户重点国有企业改革稳步推进。

　　非公有制经济继续快速发展，实现增加值1350亿元，同比增长25％。

　　铁西区振兴速度加快，产业项目和城建项目齐头并进，一个新型工业基地的形象已经展现。

　　浑南新区发展势头良好，芯片项目和南部“金廊”项目的积极推进，标志着浑南开发进入了新阶段。

　　东部汽车产业集聚迈出新步伐，完成了上通北盛二期的腾迁，启动了德国国际工业园一期建设。

　　沈阳目前已基本形成东部汽车、西部重化、南部高新技术和北部农产品加工四大工业经济布局。

　　2005年沈阳工业的高速发展将载入史册。从此，沈阳工业完全摆脱徘徊与困境，结束了在副省级城市排位靠后的局面，沈阳工业终于挺起了自己的脊梁！

　　（二）以筹备世园会为主线，城市建设和管理水平跃上新台阶

　　沈阳世界园艺博览会从2004年9月申办成功到去年底，占地246公顷的“世博园”已经完成了四大主体建筑、100个展园、园区景观和商业设施等全部工程。配套基础设施、绿化工程和商业招商也已完成。丰富多彩的文化活动项目已经确定，并正在筹备演练。

　　在世园会筹备中，仅用三个月的时间，就完成了展园的招展，创造了第一个奇迹；在不到半年的时间里，又完成了各项建设任务，创造了第二个奇迹；现在我们正在厉兵秣马，争取创造第三个奇迹，就是吸引1000万国内外宾客前来参观。这将真正实现全市人民的夙愿，“世界给沈阳一次机会，沈阳就一定能还世界一个奇迹！”

　　为了迎接世园会，我们进一步加强了城市基础设施改造和环境建设。

　　完成了大二环和青年大街改造等13项重点工程，整修小巷街路454条，道路改造面积达468万平方米，浑河胜利大桥建成通车。

　　去年共植树600万株，建设街头公共绿地100块，拆违建绿109万平方米，建成区绿化覆盖率达到40.65％，绿地率达到35.97％，人均公共绿地面积达到12平方米。完成了占地达4.2平方公里、水面积3.1平方公里的丁香湖一期工程建设。城市水面积率已由2000年的2.43％提高到去年的4.68％。

　　新建扩建7座污水处理厂，建成区新增污水处理能力35万吨／日，全市污水处理能力提高到127万吨／日，污水处理率将达到81％。投资6亿元对76公里长的细河进行了全面改造。又拆除烟囱1011根、锅炉房606处，集中供热率达到83％；完成了735辆公交车和5180辆出租车的双燃料改装工作。去年城市空气质量优良天数达到317天，比上年增加16天。全长22公里的地铁1号线已于去年11月开工建设。

　　全市开展了市容综合整治“百日行动”，对80条主要街路和4个广场，实施了立体化整治，整治力度和效果前所未有，市容市貌大为改观。

　　（三）对内对外开放取得新进展，城市国际化程度进一步加深

　　去年，我市制定出台14条政策措施，有力地推动了对外开放工作。

　　全市新批外商投资项目901个，合同外资额61.2亿美元。目前已有44户世界500强企业在我市设立了63家企业，涌现出8英寸芯片、恒隆广场等一批投资额超亿美元的重大项目。此外，全市还有爱生药业等5户企业分别在美国、加拿大、新加坡和香港资本市场上市，打破了我市多年来上市公司止步不前的局面。

　　在国家改变利用外资统计方法的情况下，全年实现利用外商直接投资21.2亿美元，同比增长106％，占全省的60％以上，达到历史最高水平。实现出口总额23.7亿美元，剔除三宝电脑特殊因素，同比增长29.7％。张士、浑南两个出口加工区和苏家屯保税物流中心的建设，将成为我市及周边城市发展进出口贸易的“内陆港”。

　　法国总理拉法兰、朝鲜总理朴凤柱、新加坡总理李显龙等外国首脑相继来访；市领导会见了来自70多个国家和地区的外宾924批9750人次，其中世界500强企业和知名跨国公司团组452批1800人次；国民党名誉主席连战、新党主席郁慕明专程访问我市。这些都表明沈阳的知名度和影响力日益彰显。

　　新开通了沈阳经上海至伦敦等4条国际航线和伊尔库茨克至沈阳定期货运航线，使沈阳的国际航线总数达到23条，进一步确立了沈阳空港在东北地区的航空枢纽地位。桃仙机场旅客吞吐量达到456万人次，同比增长9.8％；口岸出入境人数达到68.5万人次，增长15.9％。

　　先后举办了“荷兰日”等18项国际友好活动。沈阳已与国外50多个城市建立友好合作城市关系。去年我们成功举办了一系列会展活动，中欧经贸高层会议、东北亚高新技术博览会、第四届制博会和第四届韩国周等大型国际展会，都产生了广泛影响。特别是世界生产力大会的成功申办，将使沈阳大型国际活动达到新高峰。

　　一年来，全市共有689个招商团组、3457人次，分赴浙江、江苏、福建等省的60多个重点地区开展招商活动，共引进规模以上内资项目1750个，已调入资金352亿元，年内竣工项目847个。

　　（四）县和郊区经济加速发展，城市化工业化进程不断加快

　　去年四县（市）和四郊区完成生产总值885.5亿元，同比增长19％；完成固定资产投资502.9亿元，增长58.9％；实现财政收入22.7亿元，增长24.4％。这些指标不仅全部创造了历史最高水平，而且增幅居全省各县区前列。

　　去年，是沈阳历史上第三个农业丰收年，粮豆总产量完成342.7万吨，肉、蛋、奶产量分别达到70.4万吨、42.5万吨和21.5万吨，同比分别增长18.2％、12％和12.6％。

　　全市新上投资500万元以上的农产品加工项目300个，已建成各类农产品加工企业1600家，实现农产品加工业增加值118亿元。

　　市财政除每年支持4个县（市）各2000万元、2个郊区2200万元，用于基础设施建设外，又为各县和郊区村镇规划建设补贴4000万元。去年全市将14亿元软贷款用于县和郊区的基础设施建设。

　　四县（市）和四郊区工业不断取得新突破，去年实现规模以上工业总产值719.8亿元，增长72.7％。县和郊区工业的迅速发展，将从根本上解决长期困扰县区经济发展的瓶颈问题。

　　辉山农业高新区发展势头强劲。去年新引进项目195个，建区四年来，各项主要经济指标成倍增长。

　　（五）第三产业日益繁荣，城市功能进一步拓展

　　南北“金廊”和浑河两岸的开发建设，开拓了我市第三产业发展的崭新空间。去年，北部“金廊”18个重点在建项目进展顺利，并有12个新推出的地块签订了开发意向；南部“金廊”有正大金茂中心等21个标志性建筑全部规划到位，将从今年起陆续开工建设。浑河两岸共实施基础设施项目28个，以罗士圈地区为重点的浑河北岸滩地绿化工程、滩地中心广场建设、滩地体育休闲带建设、南二环道路改造已全面完成；新开工招商项目39个，总投资407.5亿元。长白地区和于洪新城开发正在加速推进，到去年末已落实项目63个。尤其是投资4.2亿元，完成了浑河南岸防洪大堤的建设，结束了浑南不设防的历史。

　　城乡商业购销两旺，新型商业业态不断发展。去年共引进500万元以上的商业项目517个，总投资额164亿元。

　　房地产投资保持平稳健康发展的态势。全年房地产开发投资完成413.6亿元，同比增长20.7％；交易面积900万平方米，交易额300亿元，同比分别增长17％和22％。

　　金融业运行平稳。年末全市金融机构本外币存款余额3697亿元，贷款余额2356亿元，同比分别增长14.1％和14.1％。继2004年韩国韩亚银行之后，去年又有韩国中小企业银行和日本东京三菱银行两家外资银行进入我市。

　　成功举办了中国沈阳国际冰雪节、清文化节和沈阳国际旅游节等大型旅游活动。全年接待国内外旅游者3532.7万人次，同比增长24.2％。旅游总收入226.3亿元，增长24.8％。

　　去年4月，辽宁中部七城市共同签署了《合作协议》，提出并着手推进了19项重点工作，由此拉开了沈阳经济区合作发展的序幕。

　　（六）坚持科学发展观，社会事业全面协调发展

　　2005年，全市实现高新技术产值1100亿元，同比增长20％。

　　图书馆、儿童活动中心、残疾人康复中心和档案馆都已全部完工并正式投入使用。

　　市政府出台了《关于进一步实施科教兴市战略，加快沈阳教育发展的若干政策措施》，作出了在农村新建100所九年一贯制学校、筹建6所万人规模的职业技术学校等决策。有39所农村九年一贯制学校已建成，22所初中改革试点校建设取得新成效。

　　我市在全国率先全面推行新型农村合作医疗制度，参加农民已达184.7万人，参合率高达88.12％。市、县财政投资2534万元改善了县医院和乡镇中心卫生院的设施条件，完善了农村三级预防保健网络。市、区两级财政安排4000万元预算资金，对城市特困居民实施医疗救助，现已有7208人享受大病医疗救助。多方筹集资金4300万元，加强了全市公共卫生应急救治和预防控制体系基础设施建设，防控高致病性禽流感工作取得阶段性胜利。

　　继续实施全民健身工程，市区两级政府共投入1029万元资金，为468个具备条件的社区安装了户外健身器材。在第十届全国运动会上，我市运动员获得奖牌总数列全省第一位。

　　养老保险扩面工作不断取得新进展，全市新增参保人员21.9万人，总计达到148万人，参保率和缴费率同比增长13％和20％；医保参保人员增加到192.6万人；失业保险参保人数增加到117.2万人，在领失业保险金人员13.49万人；全市现有城市低保救助对象8.26万户、17.07万人；农村有4.5万户、8.62万贫困人口开始享受最低生活保障。全市实现实名制就业26.7万人，发放小额担保贷款1.2亿元。

　　（七）民主法制建设不断加强，执政为民勤政廉政水平进一步提高

　　一年来，全市各级政府认真接受人大依法监督和政协民主监督，市政府主要领导坚持每季度向市人大常委会主任会议和市政协主席会议通报工作。全年办理全国、省、市人大代表建议1138件，办理省、市政协委员提案814件，办复率达到100％，满意率达到98.8％。

　　2005年，市政府共向市人大提报《沈阳市节约能源条例》等7件地方性法规草案；出台了《沈阳市公众参与环境保护办法》等13件政府规章；发布实施了《沈阳市人民政府关于禁止在浑河城区段捕鱼的通告》等15件政府通告。认真贯彻实施《行政监察法》和《行政许可法》，深入开展行政执法监督检查工作，查处违纪违法案件286件；对我市15家主要行政许可实施主体开展专项检查，共取消18项许可项目，清理出34项不符合许可法规定的收费项目，涉及资金3104万元。

　　通过开展保持共产党员先进性教育活动，积极推进了政府机关的勤政廉政建设。对全市31个国债项目的建设，90亿元国债资金的使用，以及95项市政府重大工程招投标等情况，深入开展了执法监察和行政效能监察。

　　全市各级信访部门认真做好群众来信来访接待处理工作。去年共接待群众来访21万人次，受理群众来信1.1万件次，同比分别下降32％和30％。

　　公安机关坚持开展打黑除恶，不断提高网格化巡逻的质量。全市刑事案件发案数在上年较大幅度降低的基础上，又下降了9.6％，其中杀人、抢劫等八类主要案件下降13％，街面治安案件同比下降34.4％。

　　安全生产形势平稳。各级政府多次认真组织并开展安全大检查和整改工作，去年全市共发生各类消防和安全事故7863起，同比下降9.83％；各类事故死亡857人，下降4.73％；造成直接经济损失297万元，下降29％。

　　去年，我们完成了年初确定的22件为城乡群众办实事任务。市政府集中解决涉及群众切身利益的社会难点问题，是近年来力度最大的。

　　人口与计划生育、统计、审计、物价、参事、民族、宗教、广播、电视、新闻、出版、侨务、质量技术监督、食品药品监督、气象、民兵、预备役、双拥、防震减灾、人防、档案、修志、文史、残联、红十字会和老龄工作也都取得了新成果。去年，沈阳市被授予“全国创建文明城市先进单位”和“全国民族团结进步模范集体”称号。

　　各位代表、同志们，2005年全市工作取得的成果，推动沈阳经济和社会事业发展跃上了新台阶，并由此决定了我们全面超额完成第十个五年计划。

　　过去的五年，是沈阳国民经济综合实力显著增强的五年。2005年全市实现地区生产总值2240亿元，比2000年翻了一番。五年完成固定资产投资3622.5亿元，是“九五”时期的3.6倍。2005年地方财政收入按可比口径是2000年的3.8倍。“十五”时期利用外商直接投资累计达到89.26亿美元，是“九五”时期的3.3倍。在去年全国百强城市评比中，沈阳名列第9位。这一切都充分表明，沈阳经济终于摆脱了低速徘徊的困境，进入了持续快速健康发展的轨道。

　　过去的五年，是沈阳城市面貌发生巨大变化的五年。五年共投入城建资金433亿元，把昔日烟尘笼罩、缺水少绿、污水肆虐，被世界卫生组织认定为世界十大污染城市的沈阳，一跃改造成为国家“环境保护模范城市”和国家“森林城市”。“一宫两陵”被正式列入世界文化遗产名录。今天的广大市民，都会为自己生活在这样一座蓝天碧水、绿树成阴、路宽车畅、温馨和谐的现代大都市而感到骄傲。经过五年来全市人民的艰苦奋斗，沈阳终于摆脱了“慕、马大案”的恶劣影响，城市形象和知名度大幅度提高。

　　过去的五年，是全市人民得到实惠最多的五年。我们五年为群众办实事120余件。城市居民人均可支配收入由“九五”末期的5850元提高到去年的10098元，年均增长11.5％；农民人均纯收入由3135元提高到5050元，年均增长10％；职工平均工资由6995元提高到16025元，年均增长18％；城市人均住宅建筑面积由17.4平方米提高到23.2平方米。特别是由于沈阳城市的升值，带动了房地产的升值，商品房平均价格由2000年的2546元／平方米提高到去年的3333元／平方米。

　　各位代表、同志们，五年来全市工作取得的一切成就，是在省委、省政府和市委的领导下，全市广大干部群众励精图治、努力拼搏的结果，是720万沈阳人民辛勤汗水和聪明才智的结晶。在这里，我代表市政府，向为沈阳发展作出有益奉献、付出辛勤劳动的全市各族人民致以亲切问候和崇高敬意！向给予我们支持与监督的人大代表、政协委员，各民主党派、工商联和无党派人士、人民团体，向参与沈阳发展建设的中央和省在沈单位、驻沈部队指战员、武警官兵和在沈工作的外籍人士、港澳台同胞、海外侨胞及国际友人表示衷心的感谢！

　　回顾过去的五年，我们也清醒地认识到，政府工作还面临着许多突出矛盾和问题：一是沈阳市的主要经济指标在全国副省级城市中还处于中下游水平；二是经济体制改革任务尚没有全面完成，国有企业改革还需要做最后的攻坚；三是作为区域中心城市，沈阳对周边城市乃至东北地区的辐射力还不够；四是政府职能转变还不到位，经济发展的软环境尚需进一步改善。对于上述这些问题，我们将在“十一五”期间认真加以解决。

　　二、“十一五”时期的主要任务和2006年的重点工作

　　未来五年政府工作的指导思想是：高举邓小平理论和“三个代表”重要思想伟大旗帜，全面贯彻落实科学发展观，紧紧抓住发展第一要务，以振兴老工业基地和构建和谐沈阳为主题，以改革开放和科技进步为动力，整合发展空间，拓展城市功能，做强主导产业，促进经济快速发展和社会全面进步。

　　“十一五”期间，我们要充分利用经济全球化和世界范围的产业转移，紧紧把握中央实施振兴东北老工业基地战略决策的有利时机，加快推进沈阳经济和社会事业的全面发展。到“十一五”末期，沈阳要实现三大目标：一是实现老工业基地的全面振兴；二是加快建设东北地区中心城市；三是经济总量力争进入全国副省级城市“第一集团”。

　　为此，我们将主要完成以下工作任务：

　　———经济发展实现新跨越。全市经济增长年均保持13％，力争达到15％，人均地区生产总值比2005年翻一番；地方财政收入年均增长15％，力争达到20％；社会消费品零售总额年均增长12％；利用外商直接投资年均增长15％，进出口总额年均增长20％；固定资产投资年均增长15％，力争达到20％。

　　———城市建设达到全国一流水准。为实现建成现代化国际大都市的宏伟蓝图，要按照重点空间和特色空间布局的要求，统筹规划完成奥林匹克体育中心、国际会展中心等21个标志性重大项目建设。地铁一、二号线2010年要正式投入运营，适时开工建设三、四号线，初步形成城市快速轨道交通网络。建成区绿化覆盖率要保持在40％以上；全市森林覆盖率达到36％以上；城市水面积率达到10％以上；污水处理率达到85％以上；空气质量优良天数提高到330天以上。力争各项指标接近“国家生态市”标准。

　　———产业结构更加协调合理。坚持把“工业立市”作为全市经济发展的根本方针，瞄准国际先进水平，大力发展重大技术装备、汽车及零部件等八大产业。到“十一五”末期，全市规模以上工业总产值要达到8000亿元，实现工业增加值2400亿元。以城乡协调发展为目标，继续调整农业和农村产业结构，努力建设社会主义新农村。要大力发展金融、中介、物流、旅游和房地产等现代服务业，改造提升商贸流通等传统服务业。到2010年，全市服务业增加值力争达到2000亿元以上，将沈阳逐渐建成辐射东北地区的商贸物流中心和金融中心。

　　———和谐沈阳建设达到新水平。到“十一五”末期，城市居民人均可支配收入要达到18000元，农民人均纯收入达到9000元，城市居民人均住宅建筑面积达到28平方米。社会治安综合治理得到进一步强化，社会公共安全水平全面提升，建立起较为完善的突发事件处理体系、安全监管和保障体系，以及社会利益协调和纠纷调处机制，努力构建和谐沈阳。

　　2006年是市委、市政府确定的“开放年”和“文化旅游年”。全市要坚持以对内对外开放为主线，突出工业立市和办好世园会两大重点，确保经济发展不低于上年实际增长水平，不低于沿海城市的平均增长水平，努力实现经济和社会又快又好的发展。

　　今年全市经济和社会发展的主要预期目标是：地区生产总值同比增长14％，财政收入增长15％，固定资产投资增长15％，社会消费品零售总额增长13％，利用外商直接投资增长15％，城市居民人均可支配收入增长11％，农民人均纯收入增长12％。

　　为此，要重点做好以下九项工作。

　　（一）重新整合区域空间布局，继续大力推进工业发展

　　今年经济工作的重点仍然是紧紧抓住工业不放，做到不是“工业年”，胜似“工业年”。全年要再新开工建设1000个投资规模500万元以上的工业项目，投资总额800亿元以上，规模以上工业增加值增幅达到25％以上。

　　要进一步整合区域发展空间。今年要全面启动以铁西区、于洪区、辽中县为轴线的沈西工业走廊的开发建设，一期要抓好50公里长的西部工业开发大道、装备制造业园区、化工园区和冶金园区的建设。要按照大浑南的设想，对浑河以南的浑南新区、东陵、苏家屯及和平的部分街区进行总体规划和开发建设，逐步将其建设成为新城区和新产业区。要统筹规划辉山农业高新区和新城子区的发展，全面启动沈北大开发。要全面启动棋盘山国际旅游区建设，完善总体规划，加快重大基础设施和中心服务区建设。

　　狠抓工业项目不动摇。继续全方位、多层次、宽领域地面向国内外招商，以此加速沈阳吸纳集聚资本的速度。今年全市工业要力争引进外资项目550个，投资金额达到17亿美元。

　　继续大力推进产业结构调整，转变经济增长方式，不断提高经济运行质量，努力构建节约型社会。

　　重点推进“三个一批”年销售收入超百亿元企业和产业集群的建设，使之成为沈阳未来发展的重要支撑。支持华晨、北盛、中顺等整车企业及零部件企业的建设和整合，加速壮大我市的汽车产业。

　　继续深化国有企业改革，基本完成市属重点国有大型企业股份制改造工作。

　　要放开投资领域，放大发展空间，放手支持创业，今年将召开全市推进非公有制经济发展的大会，制定更加有力的政策措施，促进非公有制经济的大发展、快发展，今年全市非公有制经济增加值要力争增长20％以上。

　　（二）深入开展好“文化旅游年”活动，举全市之力办好世园会

　　“2006中国·沈阳世界园艺博览会”是沈阳建城史上前所未有的重大展会活动，是开展“文化旅游年”活动的中心内容，是创建全国文明城市的重要载体。我们必须举全市之力，精心安排好各项活动，做到精益求精、万无一失，将这次展会办成世界园艺博览史上最成功、最精彩的一届。

　　要在全市各行各业开展“喜迎世园会，文明在沈阳”为主题的教育竞赛活动。继续巩固百日环境综合整治的成果，精雕细刻地做好城市环境的美化和绿化，向世人充分展现鲜花锦簇的美丽沈城和沈阳人民的良好精神风貌。

　　以举办世园会为契机，促进旅游业更快地发展。今年来沈旅游人数要比去年增加450万人次，同比增长13％；全市旅游业收入要争取实现265亿元，增长17％。

　　（三）推进社会主义新农村建设，加快县区工业化城市化步伐

　　要在城市总体发展框架中高起点地规划郊区和县的发展。四个郊区要分别纳入沈北大开发、大浑南、沈西工业走廊及东部旅游度假区的整体规划；四个县（市）要按照郊区进行规划。要做好重点新村镇和农村道路等基础设施的规划建设，发展壮大新产业，加速实现城镇化。

　　必须坚持把建设大项目和产业集群作为工业经济发展的重要支柱。四郊四县（市）要在年内再引进和培育4个产值超百亿元的产业集群，每个县要至少培育壮大2个龙头农业产业化项目。今年全市粮食作物面积要稳定在550万亩，粮食产量稳定在280万吨以上。

　　继续采取切实措施促进农民增收。要实施千村富民小区工程和开发式扶贫工程；继续安排资金，用于提高农业科技含量和加强对农民的职业技能培训；今年要将35万农村富余劳动力转移到非农领域，拓宽农民的增收渠道。

　　巩固农村税费改革成果。在去年的基础上，今年继续化解10％的村级债务，建立健全失地农民的合理补偿机制和安置机制。

　　要充分发挥辉山农业高新区的龙头作用，促进全市农业产业化和农业结构调整。

　　（四）围绕建设区域商贸金融中心目标，大力提升服务业整体水平

　　加快南北“金廊”和浑河两岸的开发建设。要加大南部“金廊”建设力度，陆续启动奥体中心等21个项目，其中有7个项目将在年内完成主体工程，使“金廊”成为现代服务业的聚集区和大都市形象的展示区。浑河两岸开发要充分利用滩地资源，通过广泛招商，加速建设，使其成为国内城市最长的水岸生态景观区。

　　加快对传统服务业的升级改造。加大太原街、中街、长江街、西塔等传统商业街区的改造力度，加快提升东城商业区功能水平，形成一批以新兴业态为主的特色商业街区。

　　进一步提升金融服务业的发展水平。依托沈阳金融开发区，推进区域性金融中心、金融网络体系建设，在现有5家外资银行的基础上，今年争取再引进2－3家外资银行进入我市。要进一步发展保险市场。继续推进企业在境内外资本市场上市融资。

　　推进新型工业服务体系建设，大力发展现代物流、会展和房地产业。住房消费类贷款余额要争取突破400亿元。

　　（五）抓好重大项目建设，全面拓展城市整体功能

　　要高起点规划和建设奥体中心等一批标志性建筑和重大基础设施。今年城市建设计划投资150亿元，同比增长22％。

　　要抓好地铁一号线建设，年内争取开工二号线，尽快建成十字型地下轨道交通骨架。重点建设区域性的沈康、沈新、沈通等高速公路工程。在浑河上再建设两座桥梁，增加贯通城市南北的大通道。

　　继续实施以浑河整治为主要内容的水系建设工程，建设亲水休闲区和公园，恢复和保护卧龙湖、西湖等湿地，构建环绕城市的生态水系。

　　全面启动实施生态城市建设。实施南北运河及蒲河的综合整治。要建成辉山明渠和浑南2座污水处理厂，整体改善辉山明渠、白塔堡河两岸的环境景观；改造城市排水

　　管网，关闭浑河两岸现有排污口，全市城区和“世博园”区域内的水质要全部达到国家四类标准。继续拆除一批烟囱和锅炉房，大气环境质量优良天数力争提高到320天。

　　不断巩固和提高国家“森林城市”、国家“环保模范城市”成果，建立完备的城市管理法规体系，坚持建管结合，使城市管理达到国内一流水准。

　　（六）以“开放年”活动为主题，进一步扩大对内对外开放

　　坚持以引进重大外资项目为主导，努力拓宽利用外资渠道，改进招商方式。以韩国、日本和港台地区为重点，同时强化对德国、新加坡等国家的招商引资。

　　认真解决外贸出口的瓶颈问题，出口总额要同比增长20％。沈阳出口加工区和保税物流中心要在2007年前建成。

　　进一步增进对外友好交流与合作。继续邀请更多的外国首脑和世界知名企业 C EO来我市参观考察、洽谈贸易。举办好第五届制博会和“韩国周”等重大国际展会活动。要争取再开通5条国际航线，空港出入境人数要增长30％以上。

　　对内开放仍然以江、浙、闽、粤等地区为依托，重点引进亿元以上的大项目、大企业和产业集群。

　　（七）实现科技教育事业新突破，增强城市核心竞争力

　　要以建设“国家创新型城市”为目标，把科技强市作为促进沈阳振兴的核心战略。今年将召开全市科技大会。要进一步明确方针政策，充分发挥科技人才优势，提高自主创新能力，积极推进科技创新体系建设。全市高新技术产值争取实现1380亿元，同比增长25.5％。

　　全面改造城区薄弱学校，在做好已经启动的46所初中和20所小学综合改革的基础上，再确定40所城区小学进行综合改革。在农村建设20所九年一贯制学校。年内完成100名校长、1000名教师的校际交流任务。正式启动装备制造等6所重点职业学校建设，形成技能型人才培养和输出基地，同时也为农村初中毕业生进入城市学习、就业打开通道。要采取切实措施，大力支持在沈高校资源的整合。

　　（八）推动社会事业全面发展，努力建设和谐沈阳

　　要继续扩大养老、医疗、失业、工伤等各项社会保险的覆盖面。要开展多种形式的就业培训，增加公益性岗位，全年力争实现实名制就业23.87万人，确保城镇登记失业率控制在6％以内。企业养老保险收缴比例由20％下降到19％。建立和完善失地农民的社会保障体系，抓紧制定统一规范的征地、拆迁补偿政策，切实保障失地农民的合法权益。要进一步完善城乡社会救助体系，提高低保保障水平。

　　建立健全覆盖全社会的公共文化服务体系。大力加强城乡文化基础设施建设，新建一批农村文化站和广场，丰富农民精神文化生活。全力办好第六届沈阳艺术节等大型文化艺术活动。

　　要提高公共卫生应急反应能力，建立完善的预防控制和医疗救治体系，全力做好高致病性禽流感等疫病的防控工作。要加大对食品和药品生产销售的监管力度，切实保障人民群众的饮食卫生和生命安全。积极发展城镇社区卫生服务。继续完善新型农村合作医疗制度，切实解决农民看病难的问题。

　　加强城乡体育设施建设，今年要开展5－10项具有一定规模和富有特色的群众性体育活动。

　　要加强社会公德教育，并采取行政、经济和法律等措施，营造政府、企业和市民诚信的良好环境，努力构建诚信沈阳。继续支持和发展残疾人事业。

　　进一步提高社区服务功能和水平。继续加强国防教育，不断深化双拥工作，争取沈阳连续第六次进入“全国双拥模范城市”行列。

　　要高度重视和抓好社会公共安全，全面加强安全生产和消防安全的监督管理工作，有效防止各类公共安全事故发生。切实加强社会治安综合治理，完善社会治安防控体系，坚持“网格化”巡逻等行之有效的措施，严厉打击各种刑事犯罪活动，建设平安沈阳。

　　市政府将继续大力为城乡群众办好24件实事。

　　（九）坚持依法行政，建设法治政府

　　各级政府要自觉接受人大的依法监督和政协的民主监督，认真办理人民代表的议案、建议、批评、意见和政协委员的提案。进一步畅通信访渠道，重视处理群众的来信来访。提高对突发群体性事件的快速反应和处置能力，从源头上预防和减少群体性事件的发生。

　　转变政府职能，建设法治政府。继续深化行政审批制度改革，不断提高行政审批的效率和质量。积极推行政务公开，坚持依法行政。进一步巩固保持共产党员先进性教育的成果，不断提高机关公务人员的思想和业务素质。强化执法监察和行政效能监察。坚持惩防并举、注重预防的方针，加快构建惩治和预防腐败体系，从源头上防治腐败。

　　各位代表、同志们，一个新的五年建设期开始了。在以胡锦涛同志为总书记的党中央和省委、省政府及市委的领导下，我们有“十五”期间打下的雄厚基础，有全市广大人民群众的奋力拼搏，有业已形成的良好发展态势，就一定能够再创辉煌，夺取更大的胜利！
